# Stability Study, Quantification Method and Pharmacokinetics Investigation of a Coumarin–Monoterpene Conjugate Possessing Antiviral Properties against Respiratory Syncytial Virus

**DOI:** 10.3390/ph15091158

**Published:** 2022-09-18

**Authors:** Arina G. Nemolochnova, Artem D. Rogachev, Olga P. Salnikova, Tatyana M. Khomenko, Konstantin P. Volcho, Olga I. Yarovaya, Alina V. Fatianova, Andrey G. Pokrovsky, Nariman F. Salakhutdinov

**Affiliations:** 1 N. N. Vorozhtsov Novosibirsk Institute of Organic Chemistry of the Siberian Branch of Russian Academy of Sciences, Lavrent’ev ave, 9, 630090 Novosibirsk, Russia; 2 Faculty of Natural Sciences, V. Zelman Institute for Medicine and Psychology, Novosibirsk State University, Pirogov St. 2, 630090 Novosibirsk, Russia

**Keywords:** antiviral activity, coumarin, terpene, LC-MS/MS, pharmacokinetics, RSV

## Abstract

The stability of a new coumarin derivative, agent K-142, bearing α-pinene residue and possessing antiviral activity against respiratory syncytial virus (RSV) was studied in whole mice blood in vitro, and a method for its quantification in this matrix was developed and validated. The sample preparation method was precipitation of whole blood with a mixture of 0.2 M ZnSO_4_ with MeOH (2:8 *v*/*v*) containing 2-adamantylamine hydrochloride as an internal standard (IS). Analysis was carried out by HPLC-MS/MS using reversed phase chromatography and a triple quadrupole mass spectrometer 6500 QTRAP (SCIEX) in multiple reaction monitoring (MRM) mode. The transitions 351.2 → 217.1 Da and 152.2 → 93.1/107.2 Da were monitored for K-142 and the IS, respectively. The method was validated in terms of selectivity, calibration curve, LLOQ, accuracy and precision, stability, recovery and carry over. The developed method was used for a pharmacokinetics study of the compound after its oral administration to mice at a dose of 20 mg/kg.

## 1. Introduction

The search for new antiviral agents is one of priority areas of research in medicinal chemistry. This is due to the spread of a wide range of new viral infections and emergence of new dangerous viral diseases. Respiratory syncytial virus (RSV) is one of the important causes of infant mortality [1,2]. According to the meta-analysis conducted in 2010, the number of children dying from this disease is about 200,000 annually [3]. There is no vaccine for RSV, and therapy for the infection is mainly symptomatic. Virtually the only option is the treatment with the nonspecific and ineffective antiviral drug ribavirin [4]. At the same time, recent decades have seen significant progress in the identification of possible targets for RSV and the search for low-molecular-weight inhibitors of its replication [5], giving hope for the development of effective drugs against RSV.

**Figure 1:**
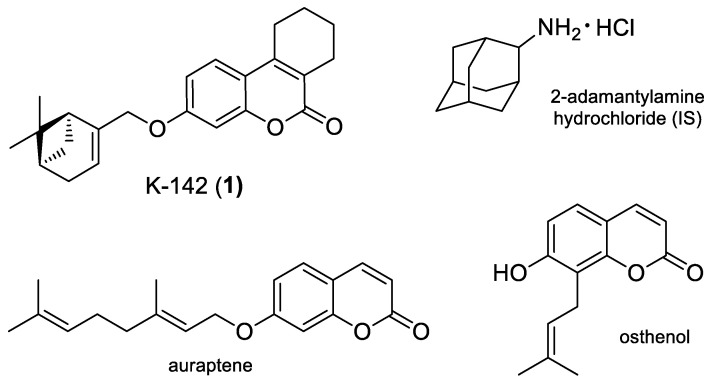
Chemical structures of K-142 (compound **1**), 2-adamantylamine hydrochloride (internal standard, IS) and bioactive coumarin derivatives auraptene and osthenol.

Prenylated coumarin derivatives are widely distributed in nature and possess a wide variety of biological activities: antitumor, neuroprotective, anti-inflammation, antiviral etc. [6,7,8]. According to Fiorito et al. [9], most interesting and perspective compounds are umbelliferone derivatives bearing geranyl and farnesyl residues. As an example, auroptene and osthenol (Figure 1) possess neuroprotective, antioxidant, cancer preventing and many other activities [10,11].

We recently discovered that a coumarin–monoterpene conjugate, agent K-142 (compound **1**, Figure 1), had high activity against RSV. This compound was active against both RSV serotype A and B (IC_50_ = 5 µM) and had high selectivity index (SI = 77) [12]. Based on the results obtained in biological experiments and molecular modeling studies, RSV F-protein was suggested as a possible target for the compound. Due to the high activity of the agent, we decided to investigate its activity in in vivo experiments. According to literature, most in vivo experiments aimed to the study of antiviral compounds are carried out in mice model due to the susceptibility of this species to viruses, e.g., influenza. According to the review [13], there are different models for studying RSV, and experiments in mice are recognized as convenient and reliable for investigation of anti-RSV compounds.

In order to investigate activity of a newly discovered bioactive substance in experiments in animals, it is necessary to select its appropriate dose and administration regimen and carry out pharmacokinetics studies. For the latter, quantification of the agent in animal blood should be performed, and, thus, an assay method is needed to investigate the compound’s stability and measure its concentration. The objective of this study was the development of an LC-MS/MS method of quantification of the agent K-142, its stability study in whole mice blood as well as its pharmacokinetics study after oral administration to mice.

## 2. Results and Discussion

### 2.1. LC-MS/MS Method Development

To develop a method for analyzing K-142 using LC-MS/MS in the MRM detection mode, a solution of the substance (1000 ng/mL) was first prepared in a mixture of methanol and water (80:20) containing formic acid 0.1% by volume. The solution was injected directly using an integrated syringe pump.

**Figure 2:**
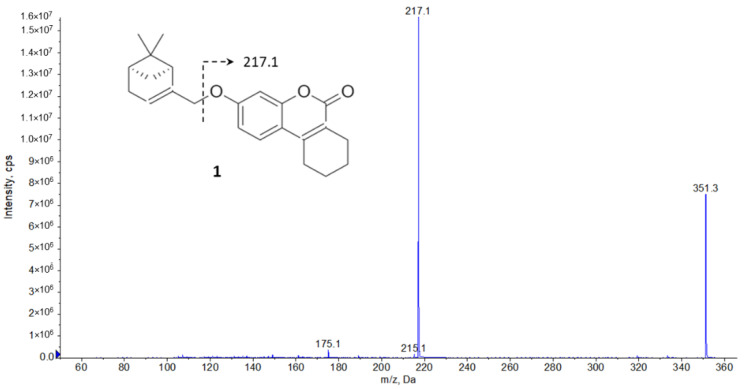
Mass spectrum of the agent K-142 (compound **1**) after collision-induced dissociation.

When the solution was infused, a molecular ion with *m*/*z* of 351.2 Da was observed in Q1 mode. When this ion, corresponding to the protonated form of K-142, was fragmented using collision-induced dissociation, a fragment ion with *m*/*z* of 217.1 Da was formed (Figure 2). No other fragment ions having similar intensity were observed in the mass spectrum while increasing the collision energy. As a result, an MRM transition of 351.2 → 217.1 was selected for the detection of K-142, and the parameters were optimized for this transition (Table S1). To study the effect of the organic component on the ionization of K-142, acetonitrile was also used to prepare a similar solution, and it was found that the peak intensity of the corresponding molecular ion was significantly lower than for the sample obtained in the MeOH–water mixture.

To optimize the chromatographic analysis conditions of K-142, a number of experiments were performed, and it was found that its elution requires a high content of the organic component of the mobile phase (98%). Despite the absence of nitrogen atoms in its composition, which usually improve ionization, K-142 gave an intense chromatographic peak, indicating the high sensitivity of the MRM method for its detection. The analysis of IS showed the applicability of the developed conditions for the analysis of both substances.

### 2.2. Stability Study of the Agent K-142 in Whole Mice Blood

Compounds containing such functional groups as esters, amides, hydroxylamine, etc., may undergo enzymatic hydrolysis in a biological matrix, primarily in blood, under the action of various enzymes such as esterases. Such hydrolysis can occur even in blood and plasma in vitro, and the rate of decomposition of substances is quite high, and the half-life can reach 10–20 min [14,15]. Low stability of an active substance in body tissues leads to a premature decrease of its amount as well as to intensification of a possible toxic effect on the body due to degradation products.

To study the stability of K-142 in whole blood of mice, a spike containing the substance at the concentration of 1000 ng/mL was prepared. After preparation, the spike was placed on a shaker and shaken throughout the experiment. Aliquots of the spike with a volume of 50 μL were taken after 5, 10, 15, 30, 60, 120, 180 and 240 min, after which the sample was prepared and analyzed, and K-142 peak area in obtained chromatograms was measured.

It can be seen from the chart (Figure S1) showing the K-142 peak area in the chromatogram that no decrease in its concentration happens for at least 4 h. Apparently, the blood enzymes do not degrade either the ether function or intramolecular ester bond.

### 2.3. Selection of the Method for Whole Blood Samples Preparation

Since verification of antiviral activity of compounds is often performed using animal models on mice, the developed method of sample preparation was aimed at minimizing the sampled blood volume, which would be 10–20 µL. For this reason, obtaining plasma from such amounts of blood is not feasible, and we aimed to develop a method for sample preparation of whole blood. The main approaches for preparation of whole blood samples of small volume are precipitation methods as well as methods based on microextraction, such as dried blood spots (DBS), volumetric adsorptive microsampling (VAMS), fabric phase sorptive extraction (FPSE) and some others [16,17].

To find the optimal method for sample preparation of mouse whole blood samples containing the agent K-142, we tested a method of protein precipitation with a mixture of 0.2 M aqueous ZnSO_4_ with MeOH (2:8). As an alternative, extraction of DBS with organic solvents with various additives was tested. Methanol, acetonitrile, ethyl acetate and methanol solutions containing 0.1% and 0.5% formic acid by volume were chosen as DBS extractants. The same mouse blood sample containing K-142 at a concentration of 100 ng/mL was used to correctly compare the efficacy of the different sample preparation methods. After preparation, the samples were analyzed, and the peaks of the substance on the chromatograms were integrated and the obtained values were used for recovery calculation. Two samples were prepared for each method, and each sample was analyzed twice.

**Figure 3:**
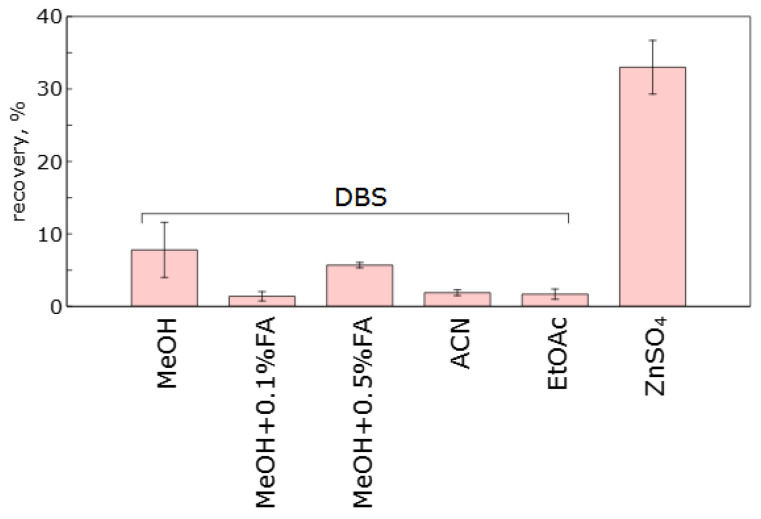
Diagram of peak area of K-142 on chromatograms of spiked samples prepared using DBS with different extracting solvents as well as whole blood mixed with the ZnSO_4_-containing precipitation solution.

Figure 3 shows the recovery values of K-142 for each sample preparation method. As can be seen from the figure, when dried blood spot is used as a sample preparation method and pure methanol is used for the extraction, there is a variation of values between repeats, which indicates a lower reproducibility of the method. Addition of formic acid (0.1% by volume) to methanol slightly reduces the recovery of the compound, but the dispersion in the results obtained is less. A further increase in the formic acid content to 0.5% by volume leads to decrease of the recovery the compound. When acetonitrile is used for extraction, the recovery was found to be very low, being about 20% of that observed for methanol containing 0.1% formic acid. Treatment of dried blood spots with ethyl acetate provides a higher extraction recovery, but this method involves evaporation of the solvent in a vacuum, which makes the overall sample preparation procedure much more complicated, especially when analyzing large sample series.

To prepare blood samples using precipitation with ZnSO_4_, 10 µL of the spike was added to 150 µL of the precipitation solution. Then, the sample obtained was stirred on a shaker, incubated, shaken again and centrifuged. The overall process took less than 10 min, which is notably shorter than the duration of DBS extraction (30 min). The obtained amount of the sample (c.a. 130 µL) allows for several analytical repeats, while the peak height was the largest in comparison with the DBS sample preparation methods tested.

As a result of the conducted experiments, precipitation of blood with a mixture of zinc sulfate with methanol was selected as a method for whole blood samples preparation, which allows for a significant decrease in the LLOQ and determines quite low concentrations of the substance. If it is not possible to perform the analysis immediately after the experiments on animals, we propose using dried blood spots for sampling followed by the extraction with methanol containing 0.1% formic acid. The latter method can be applied after validation provided that a calibration curve can be built over a required range of concentrations. In addition, the stability of the dried blood samples must be evaluated, since degradation may occur [18].

### 2.4. Method Validation

#### 2.4.1. Selectivity and LLOQ

**Figure 4:**
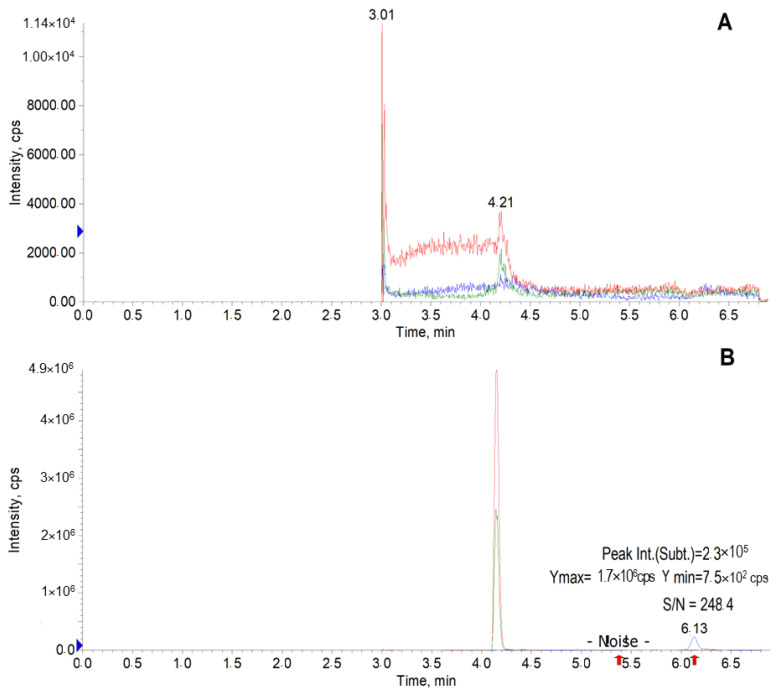
Chromatograms of a blank mice blood sample (**A**) and a mice blood sample containing K-142 at a concentration of 5 ng/mL (**B**).

Figure 4A shows a typical chromatogram of a blank blood sample obtained after sample preparation. No interfering endogenous substances eluting near the analyte or internal standard were observed. The peak of the agent K-142 in the chromatogram of a sample containing the substance at a concentration of 5 ng/mL (Figure 4B) has a signal-to-noise (S/N) ratio of 248 that meets the validation criteria (S/N ≥ 5). The retention time of the internal standard is c.a. 4.2 min, and the peak at 3.0 min (Figure 4A) is caused by the divertor valve switching.

#### 2.4.2. Calibration Curve

Standard solutions of K-142 were prepared by spiking the substance solution in methanol into mice blood at 5, 10, 20, 50, 100, 200, 500, 1000 and 2500 ng/mL concentration levels. The calibration curve with nine concentration levels was established. The calibration curve (Figure S2) was generated by plotting the peak area ratios of analyte/internal standard versus spiked analyte concentrations. The resulting calibration curve is described by the linear equation *y* = 4.94 × 10^−4^*x* + 8.87 × 10^−4^, and the correlation coefficient was calculated to be 0.9946.

#### 2.4.3. Accuracy and Precision

LLOQ, QCL and QCH samples were analyzed to assess accuracy and precision, analyses were performed on two different days. The concentrations of the agent K-142 were back-calculated using a calibration curve equation; the results obtained are shown in Table S2. CV values did not exceed 8.8% and 11.4% for intra-day and inter-day precision, respectively, and RE values for accuracy were less than 7.5%, indicating that the method was suitable for PK studies.

#### 2.4.4. Extraction Recovery and Matrix Factor

The recovery measured for QCL and QCH samples was found to be 33% and 30%, respectively. The obtained values indicate quite poor extraction of the substance from mice blood, which may be caused by its strong binding to red blood cells and/or matrix proteins. Matrix factor for QCL and QCH samples was found to be of 90.8% and 95.2%, respectively, indicating weak influence of matrix components on the analyte signal intensity.

#### 2.4.5. Carry-Over

Analysis of the ULOQ sample (the spike containing K-142 at the concentration of 2500 ng/mL) showed that the residual amount of the substance corresponded to its concentration of c.a. 0.5–0.7 ng/mL, which was less than 20% of that for LLOQ and followed the regulatory documents.

### 2.5. Pharmacokinetics Study of K-142 after Oral Administration to Mice

The method has been applied to a pharmacokinetic study of the agent K-142 in mice (*n* = 8). The substance was administered to the animals orally at a dose of 20 mg/kg. After drug administration, blood samples were taken from the tail vein and treated with the precipitating solution, and the samples were analyzed.

**Figure 5:**
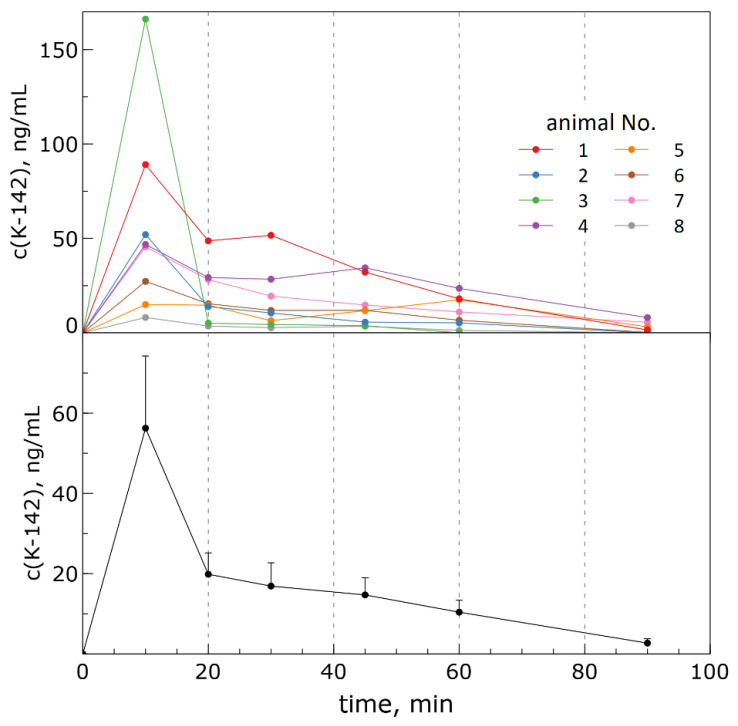
Concentration-time profiles of K-142 in blood after oral administration at a dose of 20 mg/kg in mice: (**A**) individual profiles for animals; (**B**) average profile (represented as mean values ± SEM).

Figure 5A shows the individual concentration-time profiles for each animal. It can be noted that the dependencies for the main group are similar, but those for two mice (#3 and #8 on the graph) differ from the others. Apparently, in animal #3 there was a one-step absorption of the agent followed by its rapid excretion, whereas in animal #8 there seemed to be little bioavailability of the agent. The graph representing the average pharmacokinetics profile is shown in Figure 5B.

As can be seen from the graphs in Figure 5, when the substance is administered orally, its absorption is fairly rapid, and the maximum concentration is reached 10 min after administration. The maximum values of concentrations observed for animals vary in the range from 15 to 90 ng/mL, and the average value is about 46 ng/mL. Thereafter, the content of the substance in the blood gradually decreases, and almost complete excretion is achieved in about 2 h. As the average concentration of K-142 in mice blood was c.a. 3.1 ng/mL, which is below LLOQ, this time point was not included in calculation of pharmacokinetics parameters and is shown in Figure 5 just for information. Due to the same reason, the data on the concentrations of the agent K-142 in animal #8 were also not taken into account for the calculation. The averaged values of pharmacokinetic parameters were calculated for a period of time from 0 to 60 min and are given in Table 1.

The compound under investigation, K-142, is a conjugate of a monoterpene α-pinene and coumarin, therefore, its physicochemical properties and metabolism will be determined primarily by these fragments. Thus, it is known that main metabolism of α-pinene in humans is its rapid oxidation on the CH_2_-group, resulting in the formation of corresponding diastereomeric verbenols. In addition to this, it was established that epoxidation of the double bond of the six-member cycle can occur [19]. Coumarin itself is also rapidly metabolized when administered to animals. It was shown that its major pathway is 7-hydroxylation in humans and epoxidation followed by formation of a series of hydroxylated derivatives in rats and mice [20,21]. The hydroxylated metabolites then undergo sulfation or glucuronidation and are excreted mostly with urine and feces.

Pharmacokinetics parameters calculated for K-142 are in agreement with those determined for different coumarin derivatives bearing isoprene moieties. Thus, oral administration of osthenol to ICR mice at a dose of 5 mg/kg and 20 mg/kg resulted in the following PK parameters: C_max_ of 66.1 ± 3.2 and 56.0 ± 29.1 ng/mL, respectively, and T_max_ of 36.3 ± 13.8 and 6.3 ± 1.3 min, respectively, indicating the very low bioavailability of the compound as well as its non-linear pharmacokinetics [22]. Oral administration of auraptene to rats at a dose of 100 mg/kg provided the maximum concentration of the compound in blood plasma of 1719 ± 384.3 ng/mL and T_max_ of 108.0 ± 25.3 min, and the bioavailability of auraptene was determined to be 8.5% [23]. Taking into account the data mentioned above, we suppose that the agent K-142 can be metabolized rapidly after administration to animals and metabolized in the first pass, and the search for metabolites of K-142 will be the objective of our next studies.

Thus, a method for quantification of a new perspective agent possessing anti-RSV properties was developed, validated and applied for pharmacokinetics studies of a coumarin derivative, agent K-142. The obtained results indicate that to achieve higher concentrations of the compound in animals, it is necessary to investigate its different dosage forms and possibly increase its dose.

## 3. Experimental Section

### 3.1. Chemicals and Reagents

The substance K-142 was synthesized as described in [12]. Methanol (Merck, Darmstadt, Germany) and acetonitrile (“CryoChrom”, St. Petersburg, Russia) were of HPLC grade. Formic acid was obtained from “Panreac” (Spain). Pure water was prepared using a Direct-Q 3 UV system (Millipore S.A.S., Molsheim, France). Whatman 903 Protein Saver Cards were from Sigma-Aldrich.

### 3.2. Stock Solutions, Calibrators and Quality Control Samples

To obtain stock solution of K-142 (1.0 mg/mL), a weight of the substance (c.a. 1.5 mg) was dissolved in a corresponding amount of methanol. Then, the stock solution was consequently dissolved in acetonitrile to obtain a series of working solutions having concentrations of the substance in the range from 50 ng/mL to 25 µg/mL. Aliquots of whole mice blood stabilized with EDTA (180 µL) were spiked with 20 µL of working solutions to obtain a series of calibrators and quality control (QC) samples. The concentrations of the calibrators were 5, 10, 20, 50, 100, 200, 500, 1000, 2000 and 2500 ng/mL. The concentrations of QC samples were (ng/mL): 15 (QC of low level, QCL), 1250 (QC of medium level, QCM) and 2250 (QC of high level, QCH).

### 3.3. Sample Preparation

#### 3.3.1. Dried Blood Spots Sampling and Preparation

An amount of 10 µL of a spike was spotted onto the Whatman 903 Protein Saver card and air-dried at ambient temperature for 3–4 h. For extraction, the whole spot was cut into a 0.5 mL polypropylene tube. To extract a blood spot, it was excised and put into a polypropylene tube, and 200 µL of the extracting solvent was added. The sample was shaken for 30 min, then centrifuged for 10 min at 13400 rpm (MiniSpin, Eppendorf). The supernatant was transferred into a vial and analyzed.

To extract a dried blood spot with ethylacetate, first, 100 µL of water was added to the sample, then it was shaken for 10 min, and 500 µL of EtOAc was added. The sample was then shaken for 30 min and centrifuged for 10 min at 13,400× *g* rpm to separate the layers. Then, 400 µL of the upper organic layer was pipetted into a new polypropylene tube and the solvent was evaporated in vacuo to dryness. An amount of 100 µL of methanol was then added to the residue, vortexed and centrifuged. The solution was then transferred into a vial and analyzed.

#### 3.3.2. Whole Blood Precipitation

The solution used for blood precipitation was a mixture of 2-adamantylamine hydrochloride (IS) in MeOH (100 ng/mL) and 0.2 M zinc sulfate in water (8:2, *v*/*v*). An amount of 10 µL of a blood sample was added to 150 µL of the precipitating solution; the sample was vortexed for 30 s, incubated for 5 min, then vortexed again for 30 s and centrifuged for 5 min at 13,400× *g* rpm (MiniSpin, Eppendorf). An amount of 130 µL of the supernatant was transferred into an insert in a vial and analyzed.

### 3.4. Apparatus and LC-MS/MS Conditions

A Shimadzu LC-20AD Prominence chromatograph equipped with a cooled autosampler and binary gradient pump coupled to a 6500 QTRAP mass spectrometer (SCIEX, Framingham, USA) were used for the analysis. Chromatographic separations of K-142 and IS were achieved on a ProntoSil 120-5 C18 AQ column (2 × 75 mm, 5 µm, EcoNova, Novosibirsk) with solvent A as 0.1% formic acid in water and solvent B as 0.1% formic acid in MeOH. The gradient was as follows: 0 min—5% B; 1 min—5% B; 2 min—98% B; 7 min—98% B; the flow rate was 200 µL/min; the injection volume was 10 µL.

Data acquisition was performed in MRM mode using positive electrospray ionization. The following parameters were set for the mass spectrometer: CUR (curtain gas) = 40 psi, CAD (collision-activated dissociation gas) = high, IS (ion source voltage) = 5500 V, TEM (temperature) = 250 °C, GS1 (sprayer gas) = 20 psi, GS2 (evaporator gas) = 20 psi, EP (entrance potential) = 10 V, dwell time = 80 msec. The detection parameters of K-142 and IS are given in Table S1. Analyst 1.6.3 software (AB SCIEX) was used to control the instruments, and MultiQuant 2.1 software (AB SCIEX) was used to process chromatograms.

### 3.5. Method Validation

Validation of the developed method was performed in accordance with the main regulatory documents of the FDA and EMA for the following parameters: selectivity, linearity of the calibration curve, accuracy, precision, recovery, matrix factor and stability of the analyte in the prepared sample [24,25].

#### 3.5.1. Selectivity and LLOQ

Method selectivity was evaluated by comparing chromatograms of blank blood samples taken from six mice with corresponding blood samples spiked with K-142 or IS.

The lower limit of quantification (LLOQ) was defined as the minimal concentration that could be determined with both precision (relative standard deviation, RSD) and accuracy (relative error, RE) not exceeding 20%, with an analyte signal-to-noise ratio being not less than 5.

#### 3.5.2. Linearity of Calibration Curves and Lower Limit of Quantification (LLOQ)

A least-squares regression model was used to plot the calibration curve using a weighing factor of 1/χ^2^. The calibration curve was plotted by linear fit of K-142 to IS peak area ratio against the theoretical standard concentration of the agent in the spiked sample.

#### 3.5.3. Accuracy and Precision

Precision and accuracy were calculated by analyzing low-, medium-, and high-level quality control samples. Each QC sample was freshly prepared in six replicates, and the analysis was carried out over two consecutive days. Intra- and inter-day precision are expressed by the respective coefficients of variation (CV); precision is expressed as a percentage of the mean measured concentration.

#### 3.5.4. Extraction Recovery, Matrix Factor and Prepared Samples Stability

Extraction recovery, matrix factor and samples stability were evaluated using quality control samples of low and high levels. Recovery (Rec) and matrix factor (MF) were calculated as follows:

Rec = (S_1_/S_2_) × 100(%),

MF = (S_2_/S_3_) × 100(%),

where S_1_ is the K-142 peak area in a chromatogram of a corresponding QC sample, S_2_ is the peak area of the analyte measured in a corresponding post-spiked sample and S_3_ is K-142 peak area in pure solvent (methanol) at a concentration corresponding to a maximal value, which can be obtained according to all dilution steps.

To assess the post-preparative stability, freshly prepared QC samples were analyzed and then kept in an autosampler at ambient temperature followed by repeated analysis after 24 h.

#### 3.5.5. Carry-Over

Carry-over was estimated by the analysis of a blank sample performed immediately after the analysis of the ULOQ calibrator sample (2500 ng/mL).

### 3.6. Pharmacokinetics Study

Experiments were performed on adult CD1 mice (*n* = 8) weighing 30–40 g. Animals were obtained from the animal facility of the Institute of Cytology and Genetics SB RAS and kept under standard conditions with free access to food and water. All experiments were performed in accordance with the “European Convention for the Protection of Vertebrate Animals Used for Experimental and other Scientific Purposes”, 1986.

Pharmacokinetics of the agent K-142 was studied at a dose of 20 mg/kg after its oral administration. To prepare the dosage form, a weight of the substance was mixed with c.a. 20 µL of Tween-80 followed by the dilution of the sample with physiological saline to a volume of 0.2 mL per 10 g of mouse bodyweight. Blood aliquots (10 µL) were taken in duplicate from the animal tail vein 10, 20, 30, 45 min, 1, 1.5, 2, 2.5 3, 4, 5 and 6 h after substance administration. The samples were mixed with the precipitation solution and processed according to the protocol and analyzed. Pharmacokinetic parameters were calculated using PKSolver [26] and are presented as mean ± standard error of mean (SEM).

## Figures and Tables

**Table 1:** Pharmacokinetic parameters (mean values ± SEM) of the agent K-142 after oral administration to mice (20 mg/kg).

Parameter	K-142
C_max_, ng/mL	63.4 ± 19.1
T_1/2_, min	27.7 ± 5.4
AUC_0–∞_, ng/mL×min	(1.57 ± 0.27) × 10^3^
MRT_0–∞_, min	40.5 ± 2.9
CL, (mg/kg)/(ng/mL)/min	(1.3 ± 0.2) × 10^−2^

C_max_—maximal concentration, T_1/2_—half-life, AUC_0__–∞_—area under the plasma drug concentration–time curve from zero point up to infinity, MRT_0__–∞_—mean time a molecule resides in body, CL—total clearance of drug from plasma.

## Data Availability

Data is contained within the article and supplementary material.

## Supplementary Materials

The following supporting information can be downloaded at: https://www.mdpi.com/article/10.3390/ph15091158/s1, Figure S1: Time dependence of the peak area on the chromatogram of compound K-142 in mouse whole blood samples; Figure S2: Calibration plot of the K-142-to-IS peak area ratio to concentration of K-142 in mice blood; Table S1: Multiple reaction monitoring parameters for detection of K-142 and 2-Ad (IS); Table S2: Results of precision and accuracy for determination of K-142 in quality control samples analysis.
