# Supplementary material for: Stability Study, Quantification Method and Pharmacokinetics Investigation of a Coumarin–Monoterpene Conjugate Possessing Antiviral Properties against Respiratory Syncytial Virus
Source: Pharmaceuticals (Basel). 2022 Sep 18;15(9):1158. doi: 10.3390/ph15091158 (PMC9504583; doi:10.3390/ph15091158)
Supplement: Supplementary file 1 [file pharmaceuticals-15-01158-s001.zip › pharmaceuticals-1869285-supplementary.pdf]

Supplementary materials

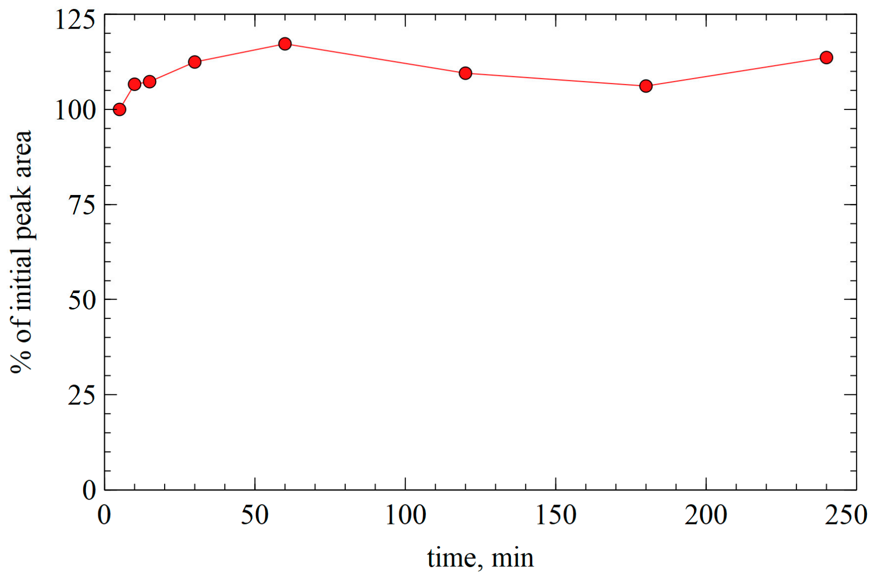

Figure S1. Time dependence of the peak area on the chromatogram of compound K-142 in mouse whole blood samples.

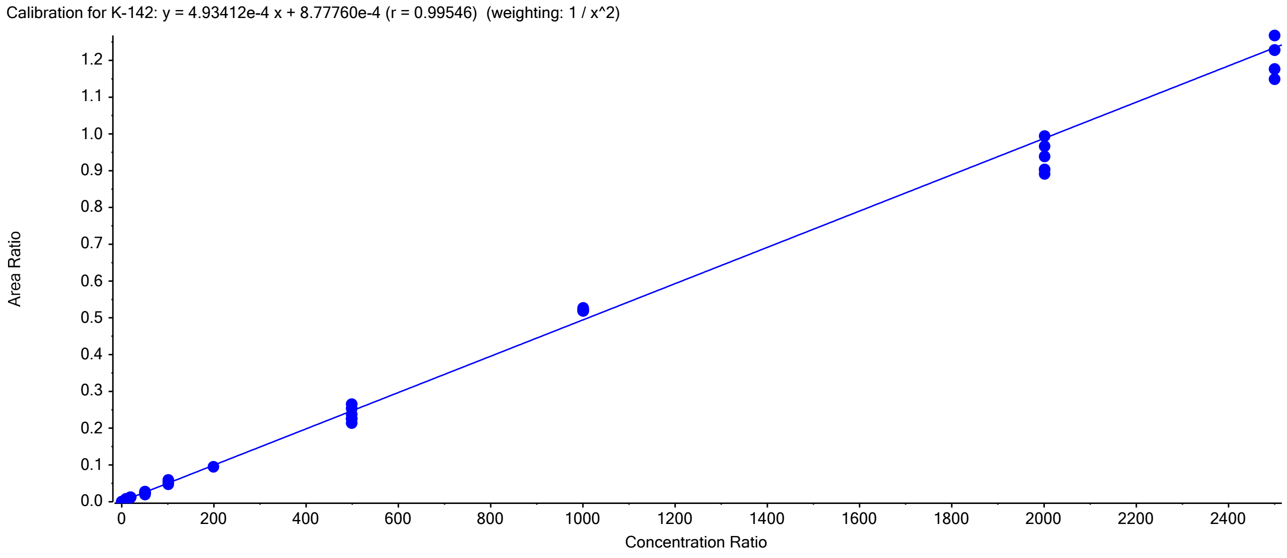

Figure S2. Calibration plot of the K-142-to-IS peak area ratio to concentration of K-142 in mice blood.

Table S1. Multiple reaction monitoring parameters for detection of K-142 and 2-Ad (IS)

Analyte and its parent ion (Q1 m/z, Da)	Product ion (Q3 m/z, Da)	DP (V)	CE (V)	CXP (V)
K-142 (351.2)	217.1	151.0	35.0	12.0
2-Ad (152.2)	93.1	16.0	35.0	14.0
	107.2	21.0	37.0	8.0

Table S2. Results of precision and accuracy for determination of K-142 in quality control samples analysis

QC sample (ng/mL)	Average found, ng/mL	Accuracy, %	CV, %
	Intra-day		
LLOQ (5)	5.28	105.6	5.1
QCL (15)	16.04	107.0	8.8
QCM (1250)	1157	92.6	3.5
QCH (2250)	2082	92.1	5.2
	Inter-day		
LLOQ (5)	5.31	106.2	5.5
QCL (15)	15.92	106.3	8.9
QCM (1250)	1225	98.1	11.4
QCH (2250)	2107	93.6	7.1
